# Quality of Life in CAR-T Cell Therapy

**DOI:** 10.3390/hematolrep18030040

**Published:** 2026-06-10

**Authors:** Caterina Alati, Martina Pitea, Gaetana Porto, Giorgia Policastro, Erica Bilardi, Giovanna Utano, Laura Giordano, Annalisa Sgarlata, Ilaria Maria Delfino, Aurora Idato, Giulia Santoro, Filippo Antonio Canale, Virginia Naso, Massimo Martino

**Affiliations:** 1 Hematology and Stem Cell Transplantation and Cellular Therapies Unit (CTMO), Department of Hemato-Oncology and Radiotherapy, Grande Ospedale Metropolitano “Bianchi-Melacrino-Morelli”, 89133 Reggio Calabria, Italy; caterina.alati@ospedalerc.it (C.A.); gaetana.porto@ospedalerc.it (G.P.); giorgia.policastro@ospedalerc.it (G.P.); erica.bilardi@ospedalerc.it (E.B.); giovanna.utano@ospedalerc.it (G.U.); giordanolau@gmail.com (L.G.); annalisa.sgarlata@ospedalerc.it (A.S.); ilariamaria.delfino@ospedalerc.it (I.M.D.); aurora.idato@ospedalerc.it (A.I.); filcan87@gmail.com (F.A.C.); virginia.naso@ospedalerc.it (V.N.); dr.massimomartino@gmail.com (M.M.); 2 Hematology, Stem Cell Transplantation, Fondazione Policlinico Universitario Campus Bio Medico, 00128 Rome, Italy; giulia.santoro@unicampus.it

**Keywords:** CAR-T, QoL, hematologic malignancies

## Abstract

**Background**: Chimeric antigen receptor T-cell (CAR-T) therapy has transformed the management of relapsed or refractory hematologic malignancies, achieving high response rates in B-cell acute lymphoblastic leukemia, diffuse large B-cell lymphoma, and multiple myeloma. While the efficacy of CAR-T therapy is well established, quality of life (QoL) metrics have become increasingly important for guiding treatment decisions, patient counseling, and survivorship planning. **Objectives**: Most patients undergoing CAR-T therapy recover their initial QoL within 3 months, an improvement not typically seen with other treatment options. A comprehensive understanding of QoL is essential for delivering patient-centered care in the evolving CAR-T landscape. **Conclusions**: This review synthesizes current evidence on QoL outcomes in CAR-T recipients, including acute effects, recovery trajectories, comparisons with conventional therapies, and strategies to optimize QoL.

## 1. Introduction

Relapsed or refractory (R/R) hematologic malignancies are among the most challenging areas in oncology [1,2,3]. Despite significant advances in conventional chemotherapy, targeted agents, and monoclonal antibodies, a substantial proportion of patients with B-cell lymphomas (BCLs), acute lymphoblastic leukemia (ALL), and multiple myeloma (MM) ultimately relapse or fail to respond to standard-of-care regimens [4,5,6,7,8]. In these heavily pre-treated populations, prognosis has historically been poor, with limited durable responses achievable through salvage chemotherapy alone.

The development of chimeric antigen receptor T-cell (CAR-T) has changed this landscape [9,10,11,12,13,14], and thousands of patients receive these therapies annually [15,16]. By harnessing and redirecting a patient’s own immune system to recognize and eliminate malignant cells, CAR-T therapy offers the potential for deep and durable remissions in patients who have exhausted conventional treatment options [17]. Since the first FDA approval in 2017 [18,19], several CAR-T products have received regulatory authorization across multiple hematologic indications, establishing this approach as a transformative pillar of modern hematologic oncology (Table 1) [20]. We have six commercially approved CAR T-cell therapy products across four disease categories, all targeting R/R hematologic malignancies. Five products target CD19 (B-cell antigens) and two target BCMA (for MM). In BCLs, three CD19-directed products are approved: axicabtageneciloleucel (AXICEL) [21,22,23,24,25,26], tisagenlecleucel (TISACEL) [27,28], and lisocabtagenemaraleucel (LISOCEL) [29,30,31,32]. Brexucabtageneautoleucel (BREXUCEL) is the key product in mantle cell lymphoma (MCL), with an impressive overall response rate (ORR) of 91% and durable PFS of 25.8 months [33,34,35], and adult ALL [36,37,38,39]. For pediatric and young adult ALL (≤25 years), TISACEL remains the only approved option [40,41]. In MM, two BCMA-targeting products are available: idecabtagenevicleucel (IDECEL) [42,43,44] and ciltacabtageneautoleucel (CILTACEL) [45,46,47,48], with CILTACEL showing the most impressive efficacy across all CAR-T products (ORR 98%, sCR 83%, median PFS 34.9 months) in the CARTITUDE-1 study [45,46].

Traditional oncologic endpoints, such as overall response rate (ORR), complete response (CR), progression-free survival (PFS), and overall survival (OS), provide essential efficacy data but do not fully capture patient experience [49,50]. Quality of life (QoL) assessment covers physical functioning, psychological well-being, social relationships, and symptom burden. It offers complementary outcome data [51,52]. These metrics are relevant for shared decision-making, expectation-setting, resource allocation, supportive care planning, development of the survivorship care model, and clinical trial design [53].

This systematic review of QoL in CAR T-cell therapy consolidates existing evidence, identifies knowledge gaps, and informs both clinical care and future research directions.

## 2. Quality-of-Life Assessment Methods

Standardized QoL measurement in CAR-T populations relies on validated patient-reported outcome (PRO) instruments [54,55] that assess domains most relevant to CAR-T survivors. Instruments such as the Functional Assessment of Cancer Therapy—Lymphoma (FACT-Lym) [56] and FACT—leukemia (Leu) [57] provide detailed evaluations of disease-specific symptoms, including fatigue- and treatment-related functional decline, which are particularly pertinent in lymphoma and leukemia populations. The European Organization for Research and Treatment of Cancer (EORTC) Quality of Life Questionnaire Core 30 (QLQ-C30) [58,59] provides a comprehensive overview of global cancer-related QOL, while the EQ-5D-5L serves as a preference-based utility measure essential for health economics analyses [60,61]. For example, FACT-Lym is more sensitive in capturing lymphoma-specific fatigue trajectories than the generic EQ-5D utility scores, enabling nuanced interpretation of patient recovery patterns. Aligning PRO tools with the domains most affected by CAR-T therapy allows for more accurate clinical assessment and supports patient-focused care planning [62].

### 2.1. FACT-Lym

The FACT-Lym is a validated, lymphoma-specific quality-of-life instrument comprising 15 items, combined with the FACT-General, and developed through provider interviews, a literature review, and patient input [56]. In 84 BCL patients, it showed good internal consistency (α = 0.79–0.85), test–retest reliability (r = 0.84), and concurrent validity with the SF-36. The scale was highly responsive to performance and treatment status, distinguishing patients who improved, remained stable, or deteriorated over time.

### 2.2. FACT-Leu

The FACT-Leu is a leukemia QoL tool developed in two phases. Items were generated by reviewing literature and interviewing 29 patients and 16 providers, yielding 17 disease-specific questions. These were added to the FACT-General to complete the tool. Testing in 79 patients showed high consistency (α = 0.75–0.96), reliable test–retest results (ICC = 0.765–0.890), good convergent validity, and response to clinical changes, supporting its use in both acute and chronic leukemia.

### 2.3. EORTC QLQ-C30

The EORTC QLQ-C30 is a validated 30-item instrument that evaluates key domains of health-related (HR) QoL. It covers emotional, physical, cognitive, social, and role functioning, as well as symptom burden, including pain, fatigue, and nausea [58,59]. The EORTC uses a modular approach: the core questionnaire is complemented by disease-specific modules, enabling a more tailored assessment of treatment impact across tumor types. Developed by the EORTC Quality of Life Group and validated across multiple cultures and languages, these instruments are now a global standard in oncology clinical trials and research. Higher scores on functioning scales indicate better functioning, whereas higher scores on symptom scales indicate greater symptom burden. This provides a nuanced and comprehensive view of the patient experience throughout cancer treatment.

### 2.4. EQ-5D-5L

The EQ-5D-5L is a standardized, generic PRO measure. It assesses HRQoL across five dimensions: mobility, self-care, usual activities, pain/discomfort, and anxiety/depression [51,60]. Each dimension is rated on five severity levels, from no problems to extreme problems. This offers greater sensitivity and reduced ceiling effects compared to the three-level version. The instrument includes a descriptive system and a Visual Analog Scale (EQ-VAS), where patients rate their health from 0 to 100. Responses generate a five-digit health state code, which can be converted into a utility score. This enables calculation of Quality-Adjusted Life Years (QALYs) for economic evaluations. Its ability to capture nuanced differences in health across conditions makes it widely adopted in clinical research and economic assessments.

Based on the evidence reviewed (Table 2), we propose a minimum core instrument approach: FACT-Lym or FACT-Leu (disease-specific) as the primary PRO instrument, supplemented by EQ-5D-5L for health-economic analyses at all time points and EORTC QLQ-C30 with appropriate modules for international or multicenter studies. Disease-specific modules and PROMIS tools may serve as optional add-ons for targeted symptom monitoring, particularly in the acute phase.

## 3. Temporal QoL Trajectory

QoL after CAR T-cell therapy usually follows a U-shaped trajectory. It initially declines within the first month due to acute toxicities such as fatigue, infections, and neurotoxicity (Table 3). Significant improvement occurs by 3 to 6 months. Most patients return to or exceed baseline functioning by 3 months. Responders experience sustained improvements in physical and mental health. However, about 20% may have persistent long-term symptoms.

### 3.1. Acute Phase (Days 0–30 Post-Infusion)

The immediate post-infusion period represents the lowest point for QoL, primarily due to anticipated toxicities and hospitalization. Cytokine release syndrome (CRS) occurs in 42% to 93% of patients, with grade 3 or higher CRS observed in 2% to 22%, depending on the CAR-T product and disease. Immune-effector-cell-associated neurotoxicity syndrome (ICANS) affects 21% to 64% of patients, with severe cases in 10% to 31%. CRS typically peaks 2 to 3 days after infusion, while ICANS peaks at 4 to 7 days. The ZUMA-1 trial was the first multicenter phase II study evaluating AXICEL in diffuse large (DL) BCL [21,22]. Among 101 treated patients, the ORR was 83% with a 58% complete response (CR) rate. After a median follow-up of over 63 months, the estimated 5-year OS rate was 42.6%, and the disease-specific survival rate was 51%. Prospective QoL data from ZUMA-1 demonstrated a meaningful decline in FACT-Lym scores at day 30 compared to baseline (mean decrease of 8 to 12 points), primarily in physical and functional well-being.

Similar trends were observed in the ELIANA [63] and KarMMa [64] studies. ELIANA was a global, phase II clinical trial that tested TISACEL in children and young adults with R/R B-cell ALL [40,41]. The trial enrolled 79 patients across 25 centers in the US, Canada, Europe, Australia, and Japan, making it the first truly global pediatric CAR-T registration trial. Results showed an ORR of around 81–82% within three months. At 12 months, roughly 59% of responders remained relapse-free. The KarMMa trials are a clinical development program evaluating IDECEL, a CAR T-cell therapy targeting the BCMA protein on myeloma cells [42,43,44,65]. The program progressed through several key studies. The original KarMMa phase 2 trial established the therapy’s effectiveness in heavily pre-treated patients, showing a 73% ORR, which led to the first-ever FDA approval of a BCMA-directed CAR-T therapy. KarMMa-3 was the first randomized phase 3 CAR-T trial in myeloma, demonstrating that IDECEL nearly tripled PFS compared to standard treatments (13.8 vs. 4.4 months). KarMMa-2 explored its use in newly diagnosed patients with suboptimal responses to frontline therapy, showing very deep responses, including a 77% CE rate.

### 3.2. Early Recovery (Months 1–3)

QoL metrics improve by month 3 for most responders. In ZUMA-1, FACT-Lym scores returned to baseline by day 90 and continued to improve in responding patients [21,22]. TRANSCEND NHL 001 was a pivotal phase 1 multicenter trial evaluating LISO-CEL, a CD19-directed CAR T-cell therapy in adults with R/R BCL. The trial enrolled 257 efficacy-evaluable patients across 14 US cancer centers, covering several aggressive BCL subtypes, including DLBCL, high-grade BCL, and primary mediastinal BCL. Results were strong, with an ORR of 73%, a CR rate of 53%, and a median OS of 27.3 months. The trial reported similar QoL recovery. Using the EORTC QLQ-C30 and EQ-5D-5L questionnaires, the authors evaluated the impact of CAR T-cell treatment on HRQoL and symptoms. Clinically meaningful improvement was observed in EORTC QLQ-C30 scores for global health status/QoL, based on a minimally important difference of 10 points at 2 months after LISOCEL infusion. There were no clinically meaningful changes in physical functioning or pain, but clinically meaningful improvements in fatigue were observed at 2 months. The proportion of patients with clinically meaningful improvement in global health status/QoL was generally higher for treatment responders than nonresponders. A trend toward decreased mean EQ-5D-5L index scores was seen at 1 month after LISO infusion, followed by increases through 18 months. Mean EQ-5D-5L visual analog scale scores increased from 2 months.

### 3.3. Long-Term Phase (≥6 Months)

Extended follow-up data show sustained improvements in QoL among durable responders. The 5-year update of ZUMA-1 found that patients in ongoing remission maintained FACT-Lym scores 10 to 12 points above baseline, with significant gains in emotional well-being [22]. Similarly, pediatric patients with ALL in ELIANA showed sustained QoL improvements, matching those of healthy age-matched controls by 12 months post-infusion among complete responders [41,66].

In the analysis based on data from the pivotal TRANSCEND trial, improvements in global health status/QoL and fatigue were maintained through 18 months after infusion, and the EQ-5D-5L VAS score tended to increase from 2 to 18 months after LISOCEL infusion [67].

## 4. CAR-T Versus Autologous-Stem-Cell Transplantation

**Figure 1:**
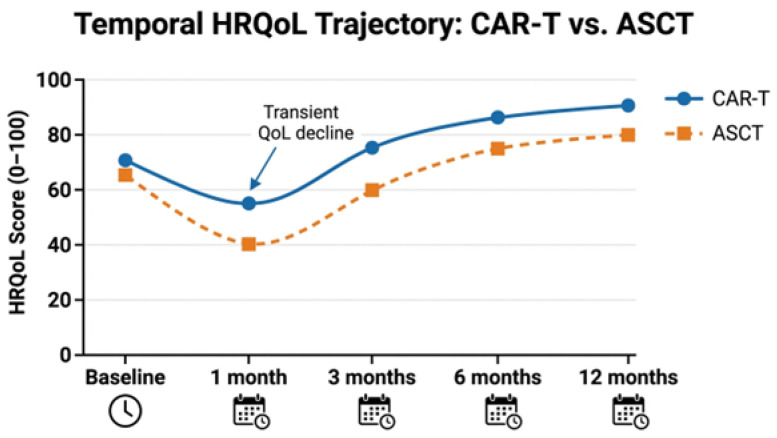
Temporal HRQoL trajectory: CAR-T vs. ASCT.

CAR-T therapy has changed the treatment of R/R BCLs, challenging the traditional role of autologous-stem-cell transplantation (ASCT) [4,68]. The temporal QoL trajectory between CAR T-cell therapy and ASCT is one of the most clinically meaningful, yet methodologically underexplored, dimensions of the comparative effectiveness debate in R/R hematologic malignancies (Table 4). Figure 1 illustrates a central dual-line graph depicting the temporal changes in HRQoL for patients undergoing CAR-T therapy versus ASCT.

Comparative QoL data from pivotal trials provide high-level evidence to guide treatment selection, especially in second-line settings. However, trial populations like those in ZUMA-7 and TRANSFORM may differ from real-world cohorts. Community settings often include patients with higher comorbidity burdens or less selective referral patterns, such as a higher Hematopoietic Cell Transplant Comorbidity Index. This could lead to delayed or less robust QoL improvements compared to clinical trial populations. These differences temper the generalizability of QoL claims and highlight the importance of considering external validity when applying these data to broader clinical practice. Of note, EU marketing authorization for all currently approved CAR-T products mandates administration exclusively at manufacturer-qualified transplant centers staffed to identify and manage early toxicity. This requirement substantially narrows the real-world versus clinical trial gap compared to other therapeutic modalities, as both settings involve highly specialized environments. Future real-world QoL studies should explicitly report center qualification status and compare outcomes between qualified centers and broader community practice where data become available.

### 4.1. Pivotal Trial Data: ZUMA-7 and TRANSFORM

The ZUMA-7 trial [23,24,69] comparing AXICEL to standard salvage chemotherapy followed by ASCT in second-line LBCL demonstrated superior PROs and improved efficacy. Using the EQ-5D-5L and FACT-Lym instruments [70,71,72], deterioration was significantly delayed with AXICEL versus standard care (HR 0.65, 95% CI 0.52–0.82). FACT-Lym scores showed earlier and sustained improvement in the CAR-T arm, with meaningful differences emerging by day 50 and persisting through 24 months; the EQ-5D visual analog scale demonstrated faster recovery to baseline and superior scores at most timepoints post-infusion.

Similarly, TRANSFORM (LISOCEL vs. standard care) [31] showed faster QoL recovery with LISOCEL (median 42 vs. 106 days to return to baseline), a higher proportion maintaining good QoL (≥90 baseline score) at 12 months (68% vs. 54%), and less chronic toxicity burden reflected in sustained functional scores.

### 4.2. BELINDA Trial: Efficacy–QoL Dissociation

The BELINDA trial (TISACEL vs. standard of care) [73] did not meet its primary efficacy endpoint but showed similar QoL profiles between treatment arms [74]. QoL was assessed using the EQ-5D-5L and PROMIS-29 questionnaires at 12 months and at a median of 36.8 months (range 15.7–57.2 months) post-cellular therapy. Groups were compared for differences in QoL indices and domain scores. Twelve months post-treatment, CAR T-cell recipients reported significantly higher QoL scores than HD-ASCT patients (EQ-5D-5 L index value: 0.89 ± 0.11 vs. 0.72 ± 0.21; *p* = 0.015). Consistently, PROMIS-29 demonstrated clinically meaningful benefits in the CAR T-cell cohort across all seven categories. At later follow-up, EQ-5D-5L index values converged (0.82 ± 0.22 vs. 0.7 ± 0.25; *p* = 0.2), whereas PROMIS-29 still indicated sustained benefit in five domains among CAR T-cell recipients. CAR T-cell therapy was associated with superior HRQoL in the first year compared to ASCT. Although some differences diminished over time, CAR T-cell patients continued to experience better outcomes in several domains. This indicates that even without efficacy advantages, CAR-T does not compromise patient-reported outcomes compared to ASCT, which is important for treatment counseling.

### 4.3. Domain-Specific QoL Considerations

CAR-T offers a faster return to baseline performance status, less prolonged neutropenia-related weakness, and lower rates of chronic pain syndromes [74]. ICANS can cause acute severe deficits but is usually reversible, with chronic cognitive impairment rare (less than 5% with persistent deficits) [75]. ASCT may lead to cumulative chemotherapy-related cognitive effects over time, though direct neurocognitive comparisons are limited [76,77].

### 4.4. Psychological Distress

Both treatments are associated with anxiety during therapy and follow-up. CAR-T patients often report uncertainty about treatment durability early on, while ASCT patients experience anticipatory anxiety about conditioning. Depression rates are similar between groups at one year [72,78,79,80].

### 4.5. Social Function

Caregiver burden is higher in the short term with CAR-T due to ICANS monitoring requirements, which often require four weeks of proximity to the treatment center [81,82]. ASCT typically requires a longer absence from work and social roles [83,84,85]. Outpatient CAR-T models can reduce disruption for some patients [86]. Geographic disparities in access to CAR-T centers also affect social and financial burdens [87].

### 4.6. Older Adults (>65 Years)

CAR-T is better tolerated than high-dose chemotherapy or ASCT, with lower treatment-related mortality. Elderly patients experience especially rapid functional recovery. In the TRANSFORM subgroup analysis [88], the QoL advantage was more pronounced in older patients.

### 4.7. Frail Patients

ASCT is often not feasible for frail patients [89]. CAR-T may be an option with careful selection, and baseline QoL and activities of daily living (ADL) status help predict recovery after CAR-T [90]. A wide variation in frailty assessment before CAR T-cell therapy is evident, with ECOG as the most commonly used tool. An appropriate frailty assessment before CAR T-cell therapy promotes the productive use of resources and proper patient selection. Using a geriatric assessment and incorporating assessment tools can help identify frail CAR T-cell therapy patients.

### 4.8. Previously Transplanted Patients

Prior ASCT does not negatively affect the CAR-T QoL trajectory [91]. Tandem transplant approaches are generally avoided due to cumulative toxicity [92].

## 5. CAR-T Versus Allogeneic-Stem-Cell Transplantation

Allogeneic-stem-cell transplantation (allo-SCT) represents an alternative curative-intent therapy for R/R disease [4,5,6,68]. Comparative QoL analyses reveal distinct temporal patterns [93,94,95]: Acute phase (0 to 3 months)—Allo-SCT recipients experience more severe and prolonged QoL declines than CAR-T recipients, due to conditioning-related toxicities, prolonged aplasia (median 14 to 21 days to neutrophil recovery), acute graft-versus-host disease (aGVHD, 30% to 50% incidence), and longer hospital stays (median 28 to 42 days versus 14 to 21 days for CAR-T). Intermediate phase (3 to 12 months)—QoL outcomes differ based on GVHD development. CAR-T recipients without disease progression generally achieve better QOL scores by month 6. Allo-HSCT recipients with chronic GVHD (cGVHD, 40% to 50% incidence) experience ongoing QoL impairment, especially in physical and social domains. Long-term phase (over 12 months)—among disease-free survivors, CAR-T recipients generally report better QoL, though both groups may face chronic health issues. Allo-SCT patients often deal with cGVHD, immunosuppression, and related complications, while CAR-T patients may experience hypogammaglobulinemia and occasional late neurotoxicity.

## 6. CAR-T Versus Salvage Chemotherapy

Elsawy et al. reported a comparative analysis of PROs with CAR-T therapy versus SOC therapy in second-line R/R LBCL from the pivotal randomized phase 3 ZUMA-7 study of AXICEL versus SOC. PRO instruments were administered at baseline, day 50, day 100, day 150, month 9, and every 3 months from randomization until 24 months or an EFS event. The QoL analysis set included patients with baseline and at least one follow-up PRO completion. Prespecified hypotheses for QoL Questionnaire-Core 30 (QLQ-C30) physical functioning, global HSQoL, and the EQ-5D-5L visual analog scale (VAS) were tested using mixed-effect models with repeated measures. Clinically meaningful changes were defined as 10 points on the QLQ-C30 and 7 points on the EQ-5D-5L VAS. Among 359 patients, 296 (AXICEL: 131; SOC: 165) met the inclusion criteria for QoL analysis. At day 100, statistically significant and clinically meaningful differences favored AXICEL for QLQ-C30 global HSQoL (estimated difference 18.1 [95% CI, 12.3–23.9]), physical functioning (13.1 [95% CI, 8.0–18.2]), and EQ-5D-5L VAS (13.7 [95% CI, 8.5–18.8]; *p* < 0.0001 for all). At day 150, scores significantly favored AXICEL for global health status/QoL (9.8 [95% CI, 2.6–17.0]; *p* = 0.0124) and EQ-5D-5L VAS (11.3 [95% CI, 5.4–17.1]; *p* = 0.0004). Responders in the CAR-T arm reported significantly better physical, emotional, and functional well-being at 12 months. In the standard care arm, QoL is affected by cumulative toxicities of sequential treatments, including salvage chemotherapy, HDC, and ASCT.

These data supported the approval of CAR-T for second-line DLBCL, with QoL benefits complementing PFS improvements. Importantly, prospective studies have shown that early PRO declines, such as a 7-point drop in the FACT-Lym score, can serve as surrogate safety signals and may predict clinical relapse in up to 68% of cases [26,96,97,98]. Such findings suggest that routine integration of PRO dashboards into clinical practice could enable earlier identification of disease progression or subacute toxicity, prompting timely intervention and improved patient management.

The phase 2 ALYCANTE trial evaluated investigator-assessed complete metabolic response at 3 months after AXICEL infusion as the primary endpoint in patients with high-risk R/R DLBCL ineligible for ASCT, showing significant improvement compared with historical controls. The trial reported HRQoL results as a secondary endpoint. HRQoL was assessed using the EORTC QLQ-C30 cancer-specific questionnaire, the QLQ-NHL-HG29, and the EuroQol EQ-5D-5L generic questionnaire at baseline and 1, 3, 6, and 12 months after CAR-T infusion. Among 62 patients, 60 (97%) completed baseline and at least one postbaseline HRQoL assessment. At 1 month, adjusted mean changes in HRQoL scores showed clinically significant deterioration in physical functioning, role, social functioning, and fatigue. However, all HRQoL dimensions recovered by 3 months and remained stable or improved by 12 months. An exploratory analysis found that adjusted mean HRQoL changes in ALYCANTE were similar to or better than in ASCT-eligible patients who received AXICEL in the phase 3 ZUMA-7 trial. Finally, global health status and fatigue scores improved to levels comparable to the general French population of similar age by 3 months. These findings indicate AXICEL improves HRQoL regardless of transplant eligibility, supporting its use across a broad patient population.

## 7. Disease-Specific QoL Considerations

While the principles of QoL apply universally, their expression differs considerably depending on the nature and trajectory of each condition. Building on this framework, the following sections explore how individual diseases impose distinct burdens on patients’ physical, emotional, and social functioning (Table 5).

### 7.1. Aggressive B-Cell Lymphomas (DLBCL, PMBCL)

Patients with aggressive lymphomas often have a high disease burden and symptoms such as bulky adenopathy, constitutional symptoms, and organ compromise [97]. Rapid disease response to CAR-T therapy often results in significant improvements in QoL, and trial data showed that physical well-being improved more than other FACT-Lym domains and correlated with reduced tumor burden [21,22,27,28,99]. Higher rates of grade 3 or 4 CRS and ICANS in DLBCL compared with indolent lymphomas are associated with more pronounced acute QOL declines [100]. However, many responders achieve a complete metabolic response by day 28, allowing relatively quick recovery of QOL.

### 7.2. Acute Lymphoblastic Leukemia

Pediatric and young adult patients with ALL in ELIANA [41,63] and ZUMA-3 [101] exhibited distinct QOL profiles. Pediatric patients often had lower baseline QOL due to multiple prior therapies, CNS disease, and the psychosocial effects of prolonged treatment. CAR-T responders showed significant improvement in both parent- and patient-reported QOL metrics by 3 to 6 months, approaching values seen in age-matched healthy controls. Notable gains included school functioning, social activities, and emotional well-being. The single-dose nature of CAR-T, compared to ongoing maintenance chemotherapy, offered a substantial QOL advantage for families [102].

### 7.3. Multiple Myeloma

Myeloma patients receiving BCMA-directed CAR-T [42,103] face unique QoL challenges, including bone disease with chronic pain, renal impairment, neuropathy from prior proteasome inhibitors, and the cumulative burden of multiple prior treatments [64,104,105,106].

The use of IDECEL in earlier lines showed significant improvements in pain interference scores by month 3 in responders (mean reduction of 1.2 points on the PROMIS Pain Interference scale) [64,104]. Physical functioning gradually improved over 6 to 12 months as bone disease stabilized. Sustained improvements in emotional well-being were associated with longer treatment-free intervals.

Rates of severe neurotoxicity were generally low (approximately 3–5% in studies) compared to other CAR T-cell indications. This safety profile, with rapid resolution of acute events, contributed to the preservation or improvement of cognitive function, which remained stable through 18 months [107].

## 8. Predictors and Modifiers of QoL Outcomes

### 8.1. Patient Profile

A consolidated assessment of baseline factors is key to predicting QoL recovery in CAR-T recipients. Three primary patient profile elements most influence QOL trajectories: performance status, age, and comorbidity burden. ECOG performance status of 2 or higher at apheresis predicts slower QOL recovery and lower peak scores. Patients with ECOG 0 or 1 experience more rapid and robust improvement. Older patients (65 or older) may have longer recovery from acute toxicities, particularly ICANS, though age alone does not prevent excellent long-term QOL in responders. Multivariable analyses from ZUMA-1 and TRANSCEND found age is an independent but modest predictor of QOL outcomes after adjusting for performance status and comorbidities. Higher Hematopoietic Cell Transplantation Comorbidity Index scores are associated with lower baseline QoL and delayed recovery. Still, disease response remains the critical determinant and may outweigh comorbidity impact in patients who achieve remission. Integrating ECOG status, age, and HCT-CI into a bundled profile streamlines risk stratification and guides recovery expectations after CAR-T therapy.

### 8.2. Treatment-Related Factors

Grade 3 or 4 CRS, especially grade 3 or 4 ICANS, significantly affect QoL trajectories. CAR-T patients show an acute decline during weeks 1 to 2, coinciding with peak CRS and ICANS toxicity. Recovery begins after 30 days, but severe toxicities, particularly ICANS, delay return to baseline QoL by 4 to 6 weeks, with persistent fatigue often lasting up to 12 months. ICANS severity strongly predicts cognitive and functional QoL at 3 months. Patients requiring ICU admission for toxicity management experience a 4- to 6-week delay in QoL recovery compared to those with grade 1 or 2 toxicities. Most grade 3 or 4 ICANS cases are reversible, with median symptom resolution in 12 days (IQR 9–15). Nearly all patients recover fully within 30 days, which is important to highlight during counseling to reduce anxiety about long-term effects.

AXICEL has higher rates of grade 3 or higher neurotoxicity, but with a rapid onset and resolution [21,22,108]. TISACEL has lower peak toxicity but may cause more prolonged CRS [27]. LISOCEL’s balanced toxicity profile in TRANSCEND was associated with less severe declines in QoL [29]. In myeloma, CILTACEL produces deeper responses than ide-cel but with higher ICANS rates; long-term QOL data suggest both products provide similar improvements in sustained responders [42,105].

### 8.3. Response Kinetics

Treatment response represents the strongest QoL predictor across all studies [54,102,109]. Complete responders consistently achieve superior QoL compared to partial responders and nonresponders. Importantly, early molecular response (undetectable disease by month 1) predicts both durable remission and optimal QoL trajectories.

Patients with disease progression often experience QoL declines one to two months before clinical or radiographic progression, highlighting the value of PROs for early detection. Routine QoL assessment may enable earlier intervention.

### 8.4. Toxicity-Related QoL Impact

Grade 1–2 CRS is related to a minimal lasting QoL impact, with most symptoms resolving by day 7 in 90% of cases. This highlights that the acute symptomatic distress is typically short-lived and does not result in durable quality-of-life consequences [75,108,110,111,112,113,114,115]. Grade 3–4 is linked to a significant acute decline but rapid recovery in most cases. Tocilizumab and steroids help reduce severity and preserve QoL outcomes.

ICANS is the most concerning acute toxicity from the patient’s perspective [107]. Grade 3–4 ICANS causes profound temporary QoL impairment, though resolution is typically complete by day 30. Persistent deficits are rare but can be devastating when they occur.

Prolonged cytopenias represent an emerging late toxicity with certain CAR-T products [116,117]. Prolonged transfusion support can significantly affect QoL, though growth factors and eltrombopag help mitigate this effect. Regarding infections, hypogammaglobulinemia occurs at comparable rates between CAR-T and transplant. The risk of opportunistic infections may be lower with CAR-T than with ASCT [118]. IVIG supplementation helps preserve QoL in all settings.

## 9. Integrating QoL Considerations into Treatment Selection

Integrating QoL considerations into treatment selection requires a systematic evaluation of patient factors, disease characteristics, and available therapies [119] (Table 6). To facilitate interactive decision-making, clinicians should discuss with patients which trade-offs are most important, such as prioritizing rapid recovery to baseline functioning versus greater certainty regarding long-term outcomes. Consideration should be given to whether a temporary decline in QoL is acceptable to achieve a quicker recovery, or whether minimizing the risk of delayed or chronic side effects is a higher priority [55]. Engaging both the care team and the patient in this reflective process enhances the relevance and individualization of shared decision-making. CAR-T QoL advantages are maximized in older patients (>65 years) who tolerate ASCT conditioning poorly, patients prioritizing rapid return to function and work/social roles, socioeconomic circumstances that favor a shorter acute treatment phase, second-line DLBCL with high-risk features (where efficacy advantages compound QoL benefits), and patients with significant baseline symptoms from disease burden [80,120]. Shared decision-making should incorporate individual tolerance for acute versus chronic toxicity patterns, work and family obligations that influence recovery timeline preferences, risk tolerance for new versus established toxicity profiles, patient values regarding the balance between efficacy and QoL, financial considerations, and the availability of caregiver support.

## 10. QoL Optimization Strategies

New strategies aim to prevent severe toxicities, and for each intervention, establishing measurable endpoints can help track real-world impacts [121]. Prophylactic tocilizumab has been shown in phase 2 studies to reduce grade 3 or higher CRS without compromising efficacy, with QoL data suggesting earlier hospital discharge and faster recovery [122]. The suggested outcome measure is a reduction in median hospital length of stay. Prophylactic corticosteroids, specifically low-dose dexamethasone, show promise in reducing neurotoxicity in high-risk patients, with a suggested outcome measure of decreased incidence of grade 3 or higher ICANS [123,124]. Anakinra (IL-1 receptor antagonist) early data suggest it may reduce neurotoxicity while maintaining anti-tumor efficacy, with a suggested outcome measure of the proportion of patients reporting improved cognitive function on validated scores at 1 month post-infusion [125]. Prompt recognition and treatment of CRS and ICANS through early intervention protocols are associated with reduced severity and improved QOL outcomes. Using validated grading scales (ASTCT consensus criteria) [126] and a low threshold for tocilizumab or corticosteroid administration has reduced the rate of grade 3 or 4 toxicity over time, as measured by the incidence of ICU admissions or grade 4 toxicity events. Selected low-risk patients may safely receive CAR-T in outpatient settings with close monitoring [86]. Typical eligibility criteria include low disease burden (LDH <2x ULN, limited bulky disease), ECOG 0–1, age < 65 years, adequate caregiver support, and geographic proximity (<1 h from treatment center). Randomized trials comparing inpatient and outpatient approaches are ongoing. For outpatients, PRO improvement (e.g., higher FACT-Lym or QLQ-C30 scores at day 30) and increased hospital-free days are recommended endpoints for future evaluation. Cognitive rehabilitation is beneficial for patients with persistent cognitive deficits after ICANS, with formal neuropsychological assessment and programs that include memory strategies, attention training, and executive function support. Early mobilization during hospitalization and structured physical therapy during recovery improve functional outcomes and physical well-being as reported by patients, with monitoring of change in the functional well-being subscale of FACT-Lym or six-minute walk distance quantifying patient benefit. Integrated psychosocial services that address anxiety, depression, and adjustment challenges can improve QOL outcomes. The unique stressors of CAR-T, including apheresis or manufacturing wait-time uncertainty, unpredictable acute toxicities, and the pressure of single-dose therapy, require specialized counseling approaches, with an outcome of reduction in patient-reported anxiety or depression scores (e.g., HADS or PHQ-9) at 3 months. Additionally, proactive financial counseling, assistance with insurance authorization, and connection to assistance programs reduce stress and improve treatment satisfaction [127], as evidenced by lower financial distress scores or improved treatment satisfaction ratings. Systematic PRO collection helps optimize clinical care and generate real-world evidence, as QOL deterioration may indicate disease progression or late toxicities that require intervention. Embedding rates of completed PRO instruments and correlation with clinical events can serve as key audit endpoints [128].

Achieving optimal QoL outcomes requires a multidisciplinary team: CAR-T physicians and advanced practice providers, neurology for ICANS management and cognitive assessment, rehabilitation medicine for functional recovery, psychiatry or psychology for mental health support, pharmacy for immunoglobulin replacement and antimicrobial prophylaxis, social work and financial navigation, and survivorship clinic integration for long-term follow-up [129]. Knowledge gaps remain regarding limited data on QoL in multiply relapsed patients receiving CAR-T beyond the third line, comparative QoL in indolent lymphomas (ASCT vs. CAR-T data emerging), long-term (>5-year) survivor QoL comparisons across modalities, optimal QoL assessment timing and instruments specific to CAR-T populations, and caregiver QoL assessments (currently underrepresented in the literature). Regional and cultural factors may substantially influence caregiver burden and available support systems. Future studies should account for national healthcare economics, cultural expectations regarding family caregiving roles, and geographic access to CAR-T centers when assessing caregiver-related outcomes. Emerging developments include outpatient CAR-T models that may further improve QoL by reducing hospitalization and enhancing patient and caregiver comfort, bispecific antibodies as additional comparators with evolving QoL data, earlier-line CAR-T use through ongoing first-line LBCL trials that may show better baseline performance status and optimize QoL outcomes, allogeneic CAR-T products with off-the-shelf availability that may change risk–benefit considerations by removing manufacturing delays, and next-generation CAR constructs including dual-targeted, armored, and controllable CARs designed to reduce toxicity while maintaining efficacy. Methodological improvements are needed to standardize PRO instruments across trials to support meta-analyses, as uniform instruments would enable future trials to deliver Level 1 QOL evidence, unlocking the potential for harmonized pooled analyses and stronger, more generalizable conclusions. Additionally, initiating real-world QoL data collection outside clinical trial settings, conducting cost-effectiveness analyses incorporating QoL weights and utility values, and developing CAR-T-specific QoL instruments that capture unique domains are essential next steps.

## 11. Controversies and Unresolved Issues

Despite the promising QoL outcomes associated with CAR-T therapy, several key controversies and unresolved issues warrant consideration. First, the generalizability of QoL advantages remains uncertain. Clinical trial populations in pivotal studies such as ZUMA-7 and TRANSFORM represent highly selected cohorts with stringent eligibility criteria, lower comorbidity burdens, and access to specialized tertiary centers. Real-world patients, who may present with higher comorbidity indices, reduced performance status, and limited access to certified CAR-T centers, may not experience equivalent QoL improvements. Second, the long-term impact of CAR-T-specific toxicities on QoL is incompletely understood. Persistent hypogammaglobulinemia requiring chronic immunoglobulin replacement, recurrent infection risk, and the rare but serious occurrence of secondary malignancies (including T-cell malignancies) represent emerging concerns in long-term survivors. How these late effects translate into sustained QoL impairment across diverse patient populations requires dedicated longitudinal studies. Third, CAR-T therapy is increasingly administered in outpatient settings in some centers; however, EU marketing authorization requirements mandate treatment at manufacturer-approved, qualified transplant centers with trained staff and early intervention protocols, due to the high-risk classification of these medicinal products. This regulatory context limits the generalizability of outpatient data and narrows the apparent gap between real-world and clinical-trial QoL outcomes compared to other treatment modalities. Finally, regional, cultural, and healthcare system differences influence both the selection and interpretation of PRO instruments. Preferences for specific tools (e.g., EQ-5D in Europe, PROMIS in the United States) reflect differing regulatory traditions, payer requirements, and patient organization priorities, making cross-regional QoL comparisons challenging. Efforts to develop CAR-T-specific, culturally validated PRO instruments and to harmonize assessment standards across trials and jurisdictions are essential for producing generalizable, Level 1 QoL evidence.

## 12. Conclusions

QoL assessment has evolved from a secondary endpoint to an essential component of CAR-T therapy evaluation. The typical QoL trajectory, characterized by acute deterioration during toxicity management, followed by recovery to baseline within 3 months and sustained improvement in durable responders, provides a framework for patient counseling and care planning. Patient-reported qualitative data consistently indicate that remission becomes meaningful only when functional recovery and well-being are restored. This perspective underscores the importance of aligning treatment efficacy with QoL at every decision point.

Comparative data increasingly show that CAR-T offers a favorable QoL profile compared to intensive alternatives, especially for patients who achieve remission. The ZUMA-7 and TRANSFORM trials provide high-level evidence for faster QOL recovery, delayed deterioration, and sustained improvements with CAR-T in second-line LBCL. Although acute toxicities such as CRS and ICANS cause temporary QoL impairment, recovery is usually rapid and complete. ASCT’s prolonged cytopenias and cumulative toxicity result in slower functional recovery, though long-term QoL among disease-free survivors becomes similar between treatments.

CAR-T is distinguished from transplantation by its single-dose administration, potential for outpatient post-acute management, and absence of chronic GVHD. Early use in second-line DLBCL is associated with better baseline functional status, thereby optimizing outcomes. Among disease-free survivors, CAR-T recipients generally report better long-term QoL than transplantation recipients.

As the CAR-T field advances, several priorities have emerged:

- Systematic PRO integration in both trials and clinical practice.
- Development of CAR-T-specific supportive care interventions.
- Comparative effectiveness research incorporating QOL as a co-primary endpoint alongside survival.
- Health economics analyses incorporating patient-centered outcomes.
- Toxicity mitigation strategies that preserve efficacy while enhancing tolerability. Patient selection should consider QoL alongside efficacy, toxicity profiles, and individual circumstances.

The move toward earlier CAR-T use will further clarify comparative QoL outcomes across disease states. As the field evolves, real-world evidence and longer follow-up will improve our understanding of how these therapies affect the overall patient experience beyond survival. Understanding and optimizing QoL in CAR-T recipients is both an ethical and practical necessity for treatment selection, resource allocation, and survivorship planning. For hematologists, QoL data complement traditional efficacy and safety metrics to support patient-centered care. Therapeutic choices should reflect not only what works, but what works best for each patient’s life.

Key clinical points:

- QoL typically nadirs at day 30 post-CAR-T infusion, returns to baseline by month 3, and improves above baseline by 6–12 months in durable responders.
- Treatment response is the strongest predictor of long-term QOL, overriding most baseline patient factors.
- Grade 3–4 ICANS particularly impacts cognitive and functional QoL domains, with recovery typically requiring 6–8 weeks.
- CAR-T generally offers superior long-term QoL compared to transplantation in disease-free survivors.
- CAR-T demonstrates faster QoL recovery compared to transplant (median 3 vs. 6–8 months) with earlier return to work and normal activities.
- Systematic PRO collection enables early detection of disease progression, late toxicities, and supportive care needs.
- Multidisciplinary supportive care—including neurology, rehabilitation medicine, and psychosocial services—optimizes QOL outcomes.
- ZUMA-7 and TRANSFORM provide Level 1 evidence supporting CAR-T’s QoL advantages in second-line LBCL.

## Figures and Tables

**Table 1:** CAR T-cell therapy: indications for relapsed/refractory hematologic malignancies.

Drug	Target	Manufacturer	FDA Indication(s)	FDA Approval	EMA Indication(s)	EMA Approval	Pivotal Trial(s)	ORR	CR Rate	Median PFS/EFS	CRS (Any/G ≥ 3)	ICANS (Any/G ≥ 3)
B-cell lymphomas												
Axicabtageneciloleucel	CD19	Kite/Gilead	R/R LBCL ≥ 2L; R/R FL ≥ 2L; R/R LBCL 2L (early relapsers)	Oct 2017 (LBCL), Mar 2021 (FL), Apr 2022 (2L)	R/R DLBCL/PMBCL/HGBCL ≥ 2L; R/R FL ≥ 3L; R/R LBCL 2L	Aug 2018, Apr 2022, Jan 2023	ZUMA-1 Ph II, single-arm; R/R LBCL ZUMA-5 Ph II, single-arm; R/R FL ZUMA-7 Ph III, RCT vs. SOC; 2L LBCL	83% 92% FL	58% 74% FL	5.9 mo LBCL; NR FLEFS HR 0.40 (Z7)	93%/9%	64%/28%
Tisagenlecleucel	CD19	Novartis	R/R ALL ≤ 25 years; R/R LBCL ≥ 2L; R/R FL ≥ 2L	Aug 2017 (ALL), May 2018 (LBCL), May 2022 (FL)	R/R ALL ≤ 25 years; R/R DLBCL/HGBCL ≥ 2L; R/R FL ≥ 2L	Aug 2018, Dec 2022 (FL)	ELIANA Ph II, single-arm; peds ALL JULIET Ph II, single-arm; R/R DLBCL ELARA Ph II, single-arm; R/R FL	81% 52% DLBCL86% FL	60% 40% DLBCL69% FL	NR (ALL 12 mo) 2.9 mo DLBCLNR FL	77%/22%	40%/13%
Lisocabtagenemaraleucel	CD19	Bristol Myers Squibb	R/R LBCL ≥ 2L; R/R LBCL 2L; R/R CLL/SLL; R/R FL; R/R MCL after BTKi	Feb 2021 (LBCL), Jun 2022 (2L), Mar 2024 (CLL/MCL/FL)	R/R DLBCL/HGBCL/PMBCL/FL3B ≥ 2L; R/R LBCL 2L	Apr 2022, Dec 2023 (2L)	TRANSCEND NHL 001 Ph I; R/R LBCL TRANSFORM Ph III, RCT; 2L LBCL TRANSCEND CLL 004 Ph I/II; R/R CLL/SLL	73% 42% CLL/SLL	53%18% CLL/SLL	6.8 mo LBCLEFS HR 0.35 (TRANSFORM)	46%/4%	30%/10%
Mantle cell lymphoma												
Brexucabtageneautoleucel	CD19	Kite/Gilead	R/R MCL after BTKi; R/R B-cell ALL (adults)	Jul 2020 (MCL), Oct 2021 (ALL)	R/R MCL after BTKi; R/R B-cell ALL (adults)	Dec 2021 (MCL), Nov 2023 (ALL)	ZUMA-2 Ph II, single-arm; R/R MCL ZUMA-3 Ph II, single-arm; adult ALL	91% 71% ALL	68% 56% ALL	25.8 mo MCL11 0.6 mo ALL	91%/18%	63%/31%
Acute lymphoblastic leukemia (ALL)												
Tisagenlecleucel	CD19	Novartis	R/R B-cell ALL in patients ≤ 25 years	Aug 2017	R/R B-cell ALL in patients ≤ 25 years	Aug 2018	ELIANA Ph II, single-arm; peds/young adult ALL	81%	60%	NR at 12 months	77%/22%	40%/13%
Brexucabtageneautoleucel	CD19	Kite/Gilead	R/R B-cell ALL in adults	Oct 2021	R/R B-cell ALL in adults	Nov 2023	ZUMA-3 Ph II, single-arm; adult R/R ALL	71%	56%	11.6 mo	91%/18%	63%/31%
Multiple myeloma												
Idecabtagenevicleucel	BCMA	BMS/2seventy bio	R/R MM ≥ 4 prior lines (IMiD + PI + anti-CD38 mAb)	Mar 2021	R/R MM ≥ 3 prior lines; refractory to last therapy	Aug 2021	KarMMa Ph II, single-arm; R/R MM KarMMa-3 Ph III, RCT vs. standard regimens	73% 71% KarMMa-3	33% sCR; 39% K-3	8.8 mo KarMMaHR 0.49 (K-3)	84%/5%	18%/3%
Ciltacabtageneautoleucel	BCMA	Janssen/Legend Biotech	R/R MM ≥ 4L; expanded ≥ 2L (lena-refractory)	Feb 2022 (≥4L), Apr 2024 (≥2L)	R/R MM ≥ 3 prior lines; refractory to last therapy	May 2022	CARTITUDE-1 Ph Ib/II, single-arm; R/R MM CARTITUDE-4 Ph III, RCT vs. SOC; 2L MM	98% 84% C-4	83% sCR; 73% C-4	34.9 mo CARTITUDE-1HR 0.26 (C-4)	95%/4%	23%/5%

FDA- and EMA-approved products—efficacy and safety from pivotal trials. CRS = cytokine release syndrome; ICANS = immune-effector-cell-associated neurotoxicity syndrome; G ≥ 3 = grade 3 or higher; ORR = overall response rate; CR = complete response; PFS = progression-free survival; EFS = event-free survival; NR = not reached; RCT = randomized controlled trial; SOC = standard of care; BTKi = BTK inhibitor; IMiD = immunomodulatory drug; PI = proteasome inhibitor; sCR = stringent complete response.

**Table 2:** Comparative summary of PRO instruments used in CAR-T QoL assessment. The following table summarizes the key characteristics, domains assessed, strengths, and recommended application scenarios for each of the four main PRO instruments used in CAR-T trials. This addition responds to the reviewer’s request for a comparative summary to assist clinicians in instrument selection and to lay the groundwork for recommending a minimum “core” instrument with optional add-ons.

Instrument	Disease Focus	No. Items	Key Domains	Strengths/Recommended Use in CAR-T
FACT-Lym	B-cell lymphomas	15 + FACT-G (27 items total)	Physical, functional, social, emotional well-being; lymphoma-specific symptoms	Most widely used in BCL CAR-T trials; captures lymphoma-specific fatigue; recommended as core instrument for BCL patients
FACT-Leu	Leukemia (ALL, CLL)	17 + FACT-G (44 items total)	Physical, functional, social, emotional well-being; leukemia-specific symptoms	Validated in both acute and chronic leukemia; recommended as core instrument for ALL/CLL patients receiving CAR-T
EORTC QLQ-C30	Pan-cancer (modular)	30	Global health status; 5 functional scales; 9 symptom scales	International standard; widely used in European CAR-T trials; use with disease-specific modules (e.g., QLQ-NHL-HG29) for added specificity
EQ-5D-5L	Generic (all conditions)	5 + EQ-VAS	Mobility, self-care, usual activities, pain/discomfort, anxiety/depression; utility scores for QALY calculation	Essential for health economic analyses; recommended as add-on to disease-specific instruments at all time points; limited sensitivity for disease-specific nuances

**Table 3:** Quality-of-life trajectory following CAR T-cell therapy.

Time Period	QOL Pattern	Primary Determinants	Mean FACT Score Change
Baseline (Pre-lympho- depletion)	Reference point	Disease burden, prior treatment toxicity, baseline performance status	Reference (0)
Day 30	Nadir (peak toxicity)	CRS/ICANS severity, hospitalization, cytopenias, infections	−8 to −15 points
Month 3	Recovery to baseline (responders)	Treatment response, toxicity resolution, immune reconstitution	−2 to +3 points
Month 6–12	Improvement above baseline	Sustained remission, reduced disease symptoms, psychological benefit	+5 to +12 points
≥12 months	Sustained improvement (durable responders)	Long-term remission, adaptation to chronic immunoglobulin needs, survivorship	+8 to +15 points

**Table 4:** Temporal QoL trajectory: CAR-T versus ASCT.

Phase	CAR-T Characteristics	ASCT Characteristics
Acute (0–3 months)	Initial QoL preserved (outpatient lymphodepletion) Acute decline if CRS/ICANS develops (days 5–14) More variable trajectory Median 2-week hospitalization	Predictable nadir days 7–14 post-conditioning Mucositis, GI toxicity dominate Gradual linear recovery Hospitalization 3–4 weeks
Subacute (3–12 months)	Cytopenias resolve faster Hypogammaglobulinemia may persist Earlier functional recovery (median 3–4 months) Lower chronic symptom burden in responders	Prolonged cytopenias common (particularly platelets) Chronic fatigue prominent Return to work/activities: median 6–8 months Infection risk with immunoglobulin dependence
Long-term (>12 months)	Less chronic health worry reported Manageable hypogammaglobulinemia No fertility concerns post-therapy QoL convergence with ASCT in durable responders	More long-term fatigue described Fertility concerns (younger patients) Cumulative toxicity burden QoL convergence with CAR-T in durable responders

**Table 5:** Disease-specific QOL patterns in CAR-T recipients.

Disease	Baseline QOL Factors	Primary QOL Gains	Recovery Timeline
DLBCL/PMBCL	Bulky disease, B symptoms, organ compromise	Rapid symptom resolution, physical functioning improvement	3–6 months
Follicular Lymphoma	Lower symptom burden, treatment fatigue from multiple prior lines	Treatment-free remission, reduced medical encounters	2–4 months
ALL (Pediatric/YA)	CNS disease, multiple relapses, psychosocial burden	School/social reintegration, cessation of maintenance therapy	3–6 months
Multiple Myeloma	Bone pain, neuropathy, renal dysfunction, heavy prior treatment	Pain reduction, treatment-free intervals, improved mobility	6–12 months

**Table 6:** Recommended QOL assessment schedule for CAR-T recipients.

Time Point	Recommended Instruments	Clinical Rationale
Baseline (pre-apheresis)	FACT-Lym/Leu, EQ-5D, PROMIS-29	Establish individual baseline for comparison; identify pre-existing deficits
Day 7, 14, 21, 30	PROMIS Global Health, symptom-specific PROs	Monitor acute toxicity impact; brief instruments appropriate for hospitalized/acutely ill patients
Month 3	FACT-Lym/Leu, EQ-5D, neurocognitive screening if prior ICANS	Assess early recovery; identify patients requiring additional supportive interventions
Month 6	FACT-Lym/Leu, EQ-5D, PROMIS-29	Key timepoint for QOL stabilization; comprehensive multidomain assessment
Month 12, then annually	FACT-Lym/Leu, survivorship-specific questionnaires	Long-term survivorship monitoring; late effect detection; survivorship care planning

## Data Availability

No new data were created or analyzed in this study.
